# Clinical and Molecular Features of Skin Malignancies in Muir-Torre Syndrome

**DOI:** 10.3390/genes12050781

**Published:** 2021-05-20

**Authors:** Dario Simic, Reinhard Dummer, Sandra N. Freiberger, Egle Ramelyte, Marjam-Jeanette Barysch

**Affiliations:** 1 Department of Dermatology, University Hospital Zurich, Raemistrasse 100, 8091 Zurich, Switzerland; dario.simic@usz.ch (D.S.); egle.ramelyte@usz.ch (E.R.); marjam.barysch-bonderer@usz.ch (M.-J.B.); 2 Faculty of Medicine, University of Zurich, Raemistrasse 71, 8006 Zurich, Switzerland; sandra.freiberger@usz.ch; 3 Department of Pathology and Molecular Pathology, University Hospital Zurich, Schmelzbergstrasse 12, 8091 Zurich, Switzerland

**Keywords:** Muir-Torre Syndrome, family history, next-generation sequencing, molecular analysis

## Abstract

Background: We investigated the mutational landscape of skin tumors in patients with Muir-Torre Syndrome (MTS) a hereditary autosomal dominant mismatch repair disorder of increased cancer susceptibility, and examined mutations other than in the DNA mismatch repair (MMR) genes. Methods: This retrospective single-center case series included seven patients with the diagnosis of Muir-Torre Syndrome with precise medical history and family history. Mutational analysis of tumor samples Formalin-fixed paraffin-embedded tissue blocks of skin lesions associated with Muir-Torre Syndrome were used for further analysis. All skin tumors were analyzed with the Oncomine Comprehensive Assay v3 (Life Technologies), which includes 161 of the most relevant cancer driver genes. Results: Eleven skin neoplasms (nine sebaceous tumors, one melanoma, one cutaneous squamous cell carcinoma) were diagnosed in seven patients. In two patients, visceral malignancies preceded the diagnosis of the skin tumors and one patient was diagnosed with a visceral malignancy after a sebaceous tumor. History of familial cancer of Lynch Syndrome (LS) was reported in three patients. The most frequently detected mutation was in the *MSH2* gene, followed by mutations in the *NOTCH1/2* and *TP53* gene. Conclusion, this study provides a molecular analysis of Muir-Torre Syndrome associated and non-associated skin tumors in patients with Muir-Torre Syndrome. Patients with sebaceous lesions should undergo microsatellite instability analysis and accurate evaluation of personal and family history to detect a possible Muir-Torre syndrome. As secondary malignancies may appear years after the first occurrence of sebaceous tumors, lifelong screening is mandatory.

## 1. Introduction

Muir-Torre Syndrome (MTS) represents a distinct variant of Lynch Syndrome (LS), previously referred to as hereditary nonpolyposis colorectal cancer (HNPCC) [1]. It is characterized as an inherited autosomal dominant disorder with early cancer susceptibility, which manifests with sebaceous adenomas or carcinomas, keratoacanthomas and tumors of visceral organs. Among visceral tumors, colorectal cancer is the most common with the prevalence of 0.5–5% [2]. Further associated malignancies are endometrial carcinoma, gastric cancer, tumors of the small bowel, transitional cell carcinoma (ureter and renal pelvis), ovarian and pancreatic tumors [3].

From a genetic perspective, all subtypes of LS underlie a microsatellite instability. This leads to an increased likelihood of the occurrence of mutations in genes, which results in oncogenic propensity [4,5]. Distinct genetic mutations are described for the MTS, most of which derive from single case studies with particular attention to mismatch repair gene mutations. So far, genetic alterations associated with MTS have been described for the DNA mismatch repair (MMR) genes *MLH1*, *MSH2*, *MSH6*, *PMS2*, *MLH3* and the non-MMR gene *EBCAM*, which epigenetically silences the closely linked *MSH2* gene [6]. Patients with *MLH1* and *MSH2* gene mutations tend to have a higher risk for malignant tumors compared to patients with a *MSH6* gene mutation [7,8,9]. In 90% of LS patients, *MLH1* and *MSH2* mutation are responsible for malignant tumors with a ratio of 1:1. Only 7–10% of the LS patients develop malignancy due to *MSH6* mutation, and 5% due to *PMS2* mutation. In contrast, 90% of the mutations in MTS occur in the *MSH2* gene, which has also shown to be responsible for more extracolonic and multiple primary cancers as compared to the *MLH1* gene [10].

We aimed to detect and compare other responsible genetic alterations in MTS associated and non-associated skin tumors in our MTS patients cohort, as sebaceous tumors with locoregional and distant metastasis of up to 14% to 25% have an aggressive potential and targeted therapy in sebaceous carcinoma has not been given much thought [11]. To address this, we collected clinical information, family history and performed next-generation sequencing (NGS)-based multiple-biomarker assay on sebaceous and non-sebaceous tumors of seven MTS patients to identify responsible mutations and to compare them to the reported data.

## 2. Materials and Methods

### 2.1. Introduction

The database (KISIM) from the University Hospital of Zurich was searched for patients with the Diagnosis of Muir-Torre Syndrome. Patients who fulfilled the diagnostic criteria from the revised Bethesda Guidelines were included into analysis [12]. Retrospective data of seven patients, including their medical and family history, were analyzed and next-generation sequencing was performed on the sebaceous and non-sebaceous skin tumors of these patients.

### 2.2. Mutational Analysis of Tumor Samples

Formalin-fixed paraffin-embedded (FFPE) tissue blocks of skin lesions associated and non-associated with MTS were used for further analysis. Representative tumor areas were marked by a dermatopathologist. Punch biopsies of 0.4 mm in diameter were taken from the corresponding areas of the FFPE blocks. DNA/RNA isolation was performed using the automated Maxwell system (Promega, Madison, WI, USA) according to the manufacturer’s manual. Library preparation was performed using 20 ng of DNA/RNA and the Oncomine Comprehensive Assay v3 (Life Technologies, Waltham, MA, USA). The Oncomine Comprehensive v3 assay was chosen as to that date it was the largest gene panel offered at our institution. The targeted genes are displayed in Table S1. It includes the most important oncogenic drivers and tumor suppressors across various types of cancers. The libraries were subsequently sequenced on the S5 sequencer (Life Technologies, Waltham, MA, USA). Data analysis was conducted using the Ion Reporter Software version 5.10 (Life Technologies, Waltham, MA, USA).

The study was conducted at the University Hospital of Zurich in Switzerland. The study protocol was approved by the institutional and regional ethical committee (KEK 2018-01565).

## 3. Results

### 3.1. Demographic Data

Seven patients were included in the analysis. The median age at diagnosis of the first MTS associated malignancy was 61 years (range 45–75 years). Five of the patients were male, two were female (Table 1).

### 3.2. Medical History

In two patients, visceral malignancies preceded the diagnosis of the skin tumors. In our cohort the most frequent visceral malignancies were urogenital tumors (two patients; 22%). One patient was diagnosed with a small lymphocytic lymphoma due to the following staging after diagnosis of a sebaceous adenoma.

### 3.3. Sebaceous Lesions and Other Cutaneous Tumors

**Figure 1:**
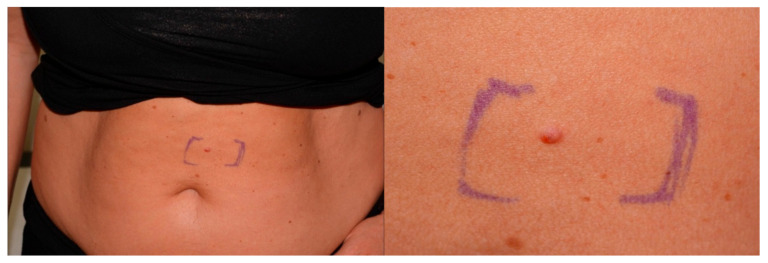
Abdominal sebaceoma patient 2. Faintly pinkish to skin-colored papule with dermoscopically yellowish ovoid areas and arborizing vessels.

**Figure 2:**
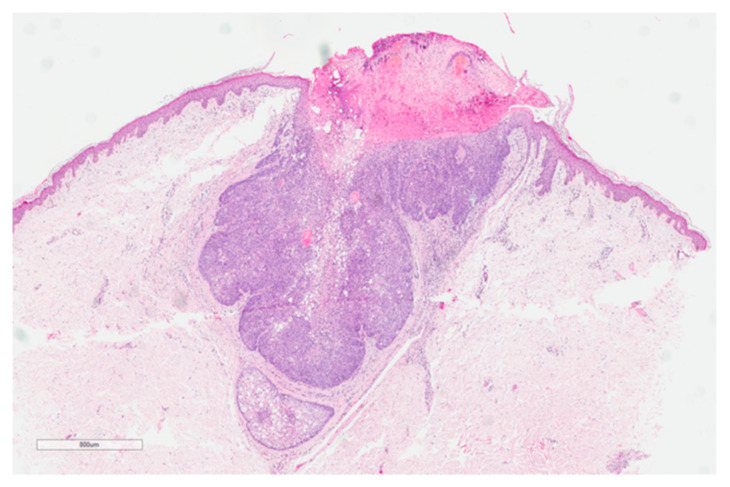
Histology of a sebaceoma abdomen patient 2 (hematoxylin and eosin stain). Circumscribed basaloid tumor with sebaceous differentiation and mitosis.

Eleven skin neoplasms were diagnosed in seven patients. Seven (64%) were located outside the head and neck region and four (36%) in the head and neck region. A photograph and histological presentation of a sebaceoma of patient 2 is illustrated in Figure 1 and Figure 2.

One patient was diagnosed with both—MTS associated and non-associated skin tumors. Additionally to a sebaceous tumors, he was diagnosed with a squamous cell carcinoma (SCC) and melanoma.

Further data are displayed in Table 1 and Table 2.

### 3.4. Family History

History of familial cancer of LS was reported in three patients. Seven of the tumors were associated with MTS and one tumor had no association with MTS.

**Figure 3:**
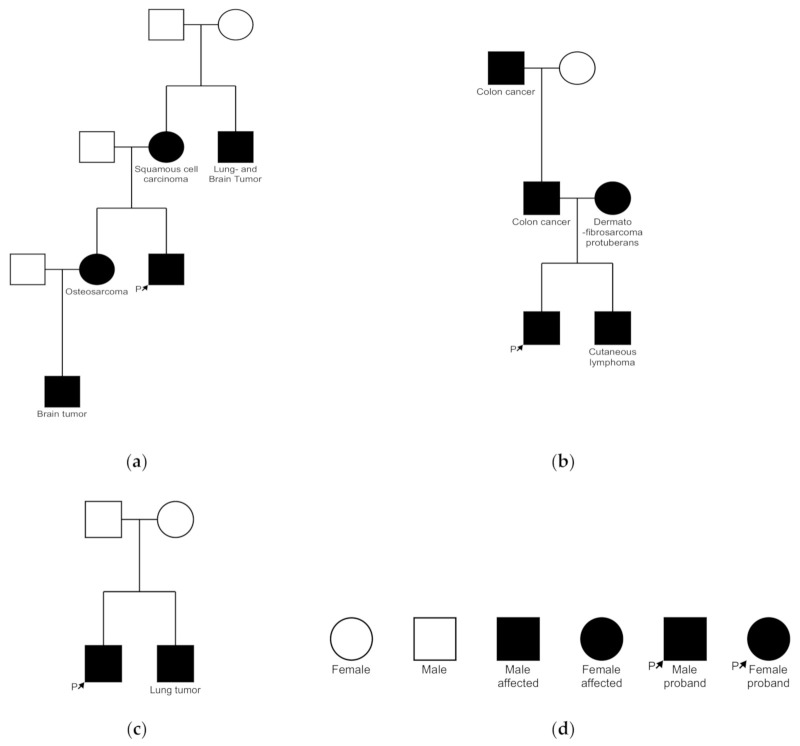
(**a**) Pedigree of patient 1 shows the autosomal dominant transmission of skin and visceral tumors; (**b**) patient 7 with MTS associated tumors in family members (**c**); patient 7 has a brother with a lung tumor; (**d**) Legend.

The pedigree of the affected families is illustrated in Figure 3.

### 3.5. Tumor Mutations

The most frequently detected mutation in all analyzed skin tumors was in the *MSH2* gene and was found in six patients (86%). The only other MMR gene mutation was *MLH1* and was only present in one patient (14%). The other most frequent mutations were in the *NOTCH1*, *NOTCH2* and in the *TP53* genes and were found in three (43%), two (29%) and two patients (29%), respectively.

The patient diagnosed with multiple sebaceous skin lesions (sebaceous carcinoma, sebaceous adenoma, tumor with sebaceous differentiation) additionally had a SCC and a melanoma in his history. All sebaceous lesions displayed *NOTCH1* and *NOTCH2* mutations. The tumor with sebaceous differentiation in this patient showed an MMR mutation in the *MLH1* gene, but this was missing in the other sebaceous tumors. The melanoma and sebaceous carcinoma showed none of the above-mentioned mutations.

The detected mutations are summarized in Table 3 and Table 4.

## 4. Discussion

### 4.1. Introduction

In this presented work, we investigated mutations other than in the MMR genes in sebaceous tumors and non-MTS associated skin lesions in patients diagnosed with Muir-Torre Syndrome. We showed which concomitant mutations may arise in these tumors, providing options for targeted therapy in advanced sebaceous tumors. With only few case reports in the literature, we furthermore examined the family histories of the affected patients, to highlight the importance of a precise medical history for a presumptive diagnosis of MTS in patients with sebaceous tumors [13,14,15].

### 4.2. Demographic Data

Sebaceous hyperplasia with frequent occurrence in the general population stands in contrast to sebaceous tumors, which are rare in general population, but often present in patients with MTS [16]. Most commonly, patients are diagnosed with sebaceous adenoma followed by sebaceous epithelioma and sebaceous carcinoma [17]. Concordant to previous data, we found sebaceous skin neoplasm in MTS patients to be more frequent outside the head and neck region.

### 4.3. Medical History

In over half of the cases, sebaceous skin lesions develop after diagnosis of a visceral malignancy, while in 22% of cases they precede visceral malignancies by up to 25 years [18,19]. This is reflected in our patient cohort with one patient being diagnosed with small lymphocytic lymphoma as he was screened for visceral malignancies after the diagnosis of a sebaceous adenoma. In two patients, visceral malignancies preceded the diagnosis of a sebaceous lesion. Apart from sebaceous tumors, squamous cell carcinoma (SSC) was reported to be associated with MMR mutations [20,21]. Additionally to sebaceous tumors, patient 1 was diagnosed with SCC and melanoma, both of which lacked MMR mutation and were most likely due to sporadic mutation. G. Ponti et al. [22] identified nine melanomas in a collective of 1057 LS patients. In only one case, *MSH2* mutation was confirmed, which indicates that the MMR genes are not a driving molecular mechanism in the genesis of melanoma, although deletions of the MMR genes can be present in sporadic melanoma [22]. One patient in our study group, was treated with cyclosporine due to a severe psoriasis vulgaris 16 years prior to the diagnosis of a solid cystic sebaceous tumor. As a switch of cyclosporine to sirolimus can decrease the frequency of sebaceous lesions, this should be considered in patients with immunosuppressive therapy [23,24].

### 4.4. Family History

We found a positive family history in three patients for visceral malignancies associated with MTS. Families with history of MTS-associated tumors, such as colorectal cancer, should undergo further genetic testing, as immunohistochemistry without family history of colorectal cancers is inferior in sensitivity and specificity as well as in the positive and negative predictive values [25]. Dermatofibrosarcoma and cutaneous T cell lymphoma (CTCL), as found in the relative of patient 2, who had a sebaceoma with a *MSH2* mutation, have been described in association with Lynch families with germline *MSH2* mutations [26]. There are also case reports of patients with CTCL, such as mycosis fungoides (MF) with MTS [27]. Scarisbrick et al. found microsatellite instability in 27% of patients with MF. Reduced expression of the hMLH1 protein was found in 56% and normal expression of the hMLH1 protein in patients without MSI, suggesting that hMLH1 promoter methylation may provide a potential future therapeutic target [28].

### 4.5. Mutational Analysis

In our collective, most commonly mutations in the mismatch repair protein (MMR) in the *MSH2* gene were detected (six patients; 86%), which is consistent with the current literature [10,29]. Besides these MMR mutations, we found diverse further mutations: The second most frequently mutated gene was *NOTCH1*, present in three patients. Reflecting the tumor suppressing function of NOTCH signaling in keratinocytes, loss of function mutations are found in SCC of the head and neck, basal cell carcinoma but also in visceral carcinomas, such as breast cancer, small cell lung carcinoma and urothelial carcinoma [30]. In contrast, gain of function mutations in the *NOTCH* genes have been described for different lymphomas [30,31,32]. Interestingly, patient 7 was diagnosed with small lymphocytic lymphoma after diagnosis of a sebaceous adenoma, which comprised mutations in *NOTCH1* and *2* additionally to the *MSH2* gene. However, mutational analysis of the bone marrow using the “TruSight Myeloid Sequencing Panel” showed no mutations. Mutations in *TP53*, coding for the tumor suppressor protein p53, for instance, was found in two patients. Yongyang Bao et al. found *TP53* mutations in 83% of the patients with sebaceous carcinoma of the eyelid [33]. This result is similar to previous studies of sebaceous carcinomas, which display a high rate for *TP53* mutations [34,35]. Patient 4 had a solid-cystic sebaceous tumor, with a *NF1* mutation. Somatic mutations in the *NF1* gene are found in a wide variety of malignant neoplasms that are not associated with Neurofibromatosis type 1 such as desmoplastic, cutaneous and mucosal melanoma and various visceral malignancies [36]. Especially in desmoplastic melanoma, *NF1* gene mutations are commonly observed with frequencies of up to 93% [37]. Tetzlaff and colleagues found two patients with *NF1* gene mutations in a collective of 27 sebaceous carcinomas [35]. An *EGFR* mutation was identified in one patient with a sebaceoma of the abdomen. *EGFR* mutations in sebaceous tumors are described in previous studies. Harvey et al. found, in a collective of 24 sebaceous lesions, nine to have an *EGFR* mutation [34]. Regarding the mutations we found in our patients collective, targeted therapy has been used with success in adenocarcinoma of the lung with *EGFR* mutations, PIK3 inhibitors in stage four melanoma or MEK inhibitors in inoperable plexiform neurofibromas with *NF1* mutations [38,39,40].

### 4.6. Diagnostic Criteria

Our findings highlight the necessity of physical examination of the skin and the lymph nodes, as well as the common screening recommendations for patients with MTS and their first-degree relatives. This includes annually physical examination (including breast in women and testicular and prostate in men), laboratory tests, colonoscopy every one to two years from the age of 25 years or five years before the youngest age of diagnosis of colorectal cancer in the family, gastroscopy and ultrasonography every one to two years [41]. As an association between sebaceous tumors and iatrogenic immunosuppression is described in the literature, particularly under the treatment of cyclosporine, patients under immunomodulatory therapy should receive regularly physical examinations and sebaceous lesions should be analyzed for microsatellite instability, as it could unmask a latent Muir-Torre phenotype [23]. In accordance with the Bethesda guidelines, patients diagnosed with colorectal cancer at an early age (<50 years), with a suggestive personal (presence of synchronous, metachronous colorectal, or other Lynch-associated tumors) or family history, should undergo further genetic testing [12].

### 4.7. Limitations

The limitation of the study is the restriction of patients number and tumor samples’ origin. Furthermore, the evaluation of the precise mutations being responsible for the visceral tumors in our patients collective would be important to draw a connection to the skin tumors. As only skin samples were analyzed for MMR mutations, and we did not look for germline mutations, we cannot rule out that the here described mutations are somatic. Germline mutations in the MMR genes could provide information about the cause of the tumors. Further molecular analysis of the tumors in the patients’ relatives or genetic testing of normal tissue or blood of the patients could give an indication of possible inherited mutations causing the Muir-Torre syndrome in this family.

## 5. Conclusions

This study provides an extensive molecular analysis of MTS associated and non-associated skin tumors in patients with MTS. We showed how different MTS-associated tumors can appear throughout generations in patients with mutations in the *MSH* genes. Therefore, along with MSI analysis, accurate evaluation of personal and family history is mandatory for patients with sebaceous lesions to detect a possible Muir-Torre syndrome. As secondary malignancies may appear years after the first occurrence of sebaceous tumors, lifelong screening is required. Furthermore, the importance of physical examination of the skin is undeniable.

## Figures and Tables

**Table 1:** Demographic data.

Total Number of Patients	7
Male	5
Female	2
Age (range)	61 (45–75)
Localization	
Non- head and neck	7
Head and neck	4
Tumor type (total number)	11
Sebaceous adenoma	2
Sebaceoma	1
Solid-cystic sebaceous neoplasm	1
Sebaceous carcinoma	4
Tumor with sebaceous differentiation	1
Squamous cell carcinoma	1
Melanoma	1

**Table 2:** Patients data.

Patient	Sebaceous Tumor (Age at Diagnosis)	Visceral Tumor (Age at Diagnosis)	Family History	Localization
1	1. Sebaceous carcinoma (63) 2. Tumor with sebaceous differentiation (63) 3. Sebaceous adenoma (67)	Caecal carcinoma (44) Bladder carcinoma (63) Adenocarcinoma sigma (67)	Sister: Osteosarcoma Uncle (mother’s side): Lung- and brain tumor Sisters’ son: Brain tumor	1. Thorax 2. Forehead 3. Back
2	Sebaceoma (55)	-	Brother: Cutaneous lymphoma Mother: Dermatofibrosarcoma Father and grandfather: Colorectal carcinoma	Abdomen
3	Sebaceous carcinoma (63)	Renal cell carcinoma (58)	-	Hip
4	Solid-cystic sebaceous tumor (62)	-	-	Back
5	Sebaceous carcinoma (65)	-	-	Back
6	Sebaceous carcinoma (75)	-	-	Back
7	Sebaceous adenoma (72)	Small lymphocytic lymphoma	Brother: Lung tumor	Cheek

**Table 3:** Most frequent mutations.

Mutated Gene	*MSH2*	*MLH1*	*NOTCH1*	*NOTCH2*	*TP53*
Total number (%)	6 (86%)	1 (14%)	3 (43%)	2 (29%)	2 (29%)
Histological type					
SC	4	-	2	2	1
SCS	1	-	1	-	1
SE	1	-	-	-	-
SA	-	1	-	-	-
Localization					
Non-head and neck	5	1	2	1	2
Head and neck	1	-	1	1	-

Abbreviations: SC, sebaceous carcinoma; SCS, solid-cystic sebaceous neoplasm; SE, sebaceoma; SA, sebaceous adenoma.

**Table 4:** Patient’s collective and mutational burden.

P	Sex (m/f)	Tumor Type (Tumor Cell Content)	Locus	Genes	% Frequency	Amino Acid Change	Coverage	Exon	Transcript	Coding	ACMG Class
1	m	SC (80%)	chr1:120462204	NOTCH2	42	p.(Arg1838*)	2000	31	NM_024408.3	c.5512C>T	Pathogenic (class 5)
			chr5:67591079	PIK3R1	46	p.(Glu558fs)	1992	13	NM_181523.2	c.1674_1675del	Uncertain significance (class 3)
			chr9:139404265	NOTCH1	45	p.(Cys963*)	1985	18	NM_017617.4	c.2889C>A	Likely pathogenic (class 4)
			chr9:139405616	NOTCH1	44	p.(Thr859fs)	1923	16	NM_017617.4	c.2574_2575ins	Likely pathogenic (class 4)
		TDS (70%)	chr1:120491661	NOTCH2	41	p.(Ser856fs)	1623	16	NM_024408.3	c.2566_2567del	Likely pathogenic (class 4)
			chr3:37090403	MLH1	43	p.(Trp666*)	2000	18	NM_000249.3	c.1998G>A	Pathogenic (class 5)
			chr3:47103763	SETD2	40	p.(Asp2064fs)	1043	14	NM_014159.6	c.6181_6182del	Uncertain significance (class 3)
			chr9:139397730	NOTCH1	39	p.(Gln1691*)	810	27	NM_017617.4	c.5071C>T	Pathogenic (class 5)
			chr10:123279674	FGFR2	35	p.(Pro253His)	1677	7	NM_000141.4	c.758C>A	Likely pathogenic (class 4)
			chr16:3646385	SLX4	5	p.(Gln565*)	519	8	NM_032444.3	c.1693C>T	Pathogenic (class 5)
		M (50%)	chr16:2135234	TSC2	5	p.(Gln1525*)	366	36	NM_000548.4	c.4573C>T	Pathogenic (class 5)
		SA (80%)	chr1:120464352	NOTCH2	9	p.(Gln1765fs)	1939	29	NM_024408.3	c.5293delC	Likely pathogenic (class 4)
		SCC (70%)	chr9:139417303	NOTCH1	13	p.(Pro247fs)	1007	4	NM_017617.4	c.740_741ins	Likely pathogenic (class 4)
			chr4:153249385	FBXW7	22	p.(Arg465Cys)	2000	9	NM_033632.3	c.1393C>T	Likely pathogenic (class 4)
			chr17:37687555	CDK12	27	p.(Gly1487*)	2000	14	NM_016507.3	c.4459G>T	Uncertain significance (class 3)
			chr17:37879658	ERBB2	30	p.(Arg678Gln)	1999	17	NM_004448.3	c.2033G>A	Uncertain significance (class 3)
2	f	SE (70%)	chr2:47698134	MSH2	42	p.(Asn566fs)	1907	11	NM_000251.2	c.1697del	Pathogenic (class 5)
			chr7:55241677	EGFR	16	p.(Glu709Lys)	1997	18	NM_005228.4	c.2125G>A	Uncertain significance (class 3)
3	m	SC (80%)	chr2:47630386	MSH2	59	p.(Phe22fs)	1835	1	NM_000251.2	c.62_63ins	Pathogenic (class 5)
4	m	SCS (80%)	chr2:47637361	MSH2	42	p.(Tyr165*)	1999	3	NM_000251.2	c.495T>A	Pathogenic (class 5)
			chr3:178952085	PIK3CA	36	p.(His1047Arg)	1658	21	NM_006218.3	c.3140A>G	Pathogenic (class 5)
			chr9:139418362	NOTCH1	25	p.(Asn70fs)	4684	3	NM_017617.4	c.209_210ins	Likely pathogenic (class 4)
			chr9:139418364	NOTCH1	40	p.(Asn70fs)	1996	3	NM_017617.4	c.207_208ins	Likely pathogenic (class 4)
			chr10:89711899	PTEN	46	p.(Arg173Cys)	1797	6	NM_000314.6	c.517C>T	Likely pathogenic (class 4)
			chr12:133202239	POLE	17	p.(Gln2217*)	128	47	NM_006231.3	c.6649C>T	Pathogenic (class 5)
			chr17:7578403	TP53	39	p.(Cys176Tyr)	1998	5	NM_000546.5	c.527G>A	Likely pathogenic (class 4)
			chr17:29665125	NF1	43	p.(Gln2263*)	2000	45	NM_001042492.2	c.6787C>T	Pathogenic (class 5)
5	f	SC (60%)	chr2:47693918	MSH2	42	p.(Gln545*)	144	10	NM_000251.2	c.1632_1633del	Pathogenic (class 5)
6	m	SC (70%)	chr2:47693799	MSH2	36	p.(Pro507fs)	2283	10	NM_000251.2	c.1518_1519ins	Pathogenic (class 5)
			chr2:47693804	MSH2	72	p.(Pro507fs)	1200	10	NM_000251.2	c.1518_1519ins	Pathogenic (class 5)
			chr16:23637644	PALB2	4	p.(Ile887fs)	637	7	NM_024675.3	c.2659_2660del	Uncertain significance (class 3)
7	m	SA (30%)	chr1:120466424	NOTCH2	40	p.(Arg1567fs)	1944	26	NM_024408.3	c.4694_4695ins	Likely pathogenic (class 4)
			chr2:47698188	MSH2	82	p.(Asn583fs)	1984	11	NM_000251.2	c.1747_1748del	Pathogenic (class 5)
			chr9:139402690	NOTCH1	41	p.(Arg1107*)	901	20	NM_017617.4	c.3319C>T	Pathogenic (class 5)
			chr11:534289	HRAS	43	p.(Gly12Ser)	1188	2	NM_001130442.2	c.34G>A	Uncertain significance (class 3)

Abbreviations: P, patient number; SC, sebaceous carcinoma; TSD, tumor with sebaceous differentiation; M, melanoma; SA, sebaceous adenoma; SCC, squamous cell carcinoma; SE, sebaceoma; SCS, solid-cystic sebaceous neoplasm.

## Data Availability

Not applicable.

## Supplementary Materials

The following are available online at https://www.mdpi.com/article/10.3390/genes12050781/s1, Table S1: List of gene targets in the Oncomine Comprehensive Assay v3. (Thermo Fisher Scientific, Waltham, MA, USA)—161 gene panel.
