# Supplementary material for: Clinical and Molecular Features of Skin Malignancies in Muir-Torre Syndrome
Source: Genes (Basel). 2021 May 20;12(5):781. doi: 10.3390/genes12050781 (PMC8160778; doi:10.3390/genes12050781)
Supplement: Supplementary file 1 [file genes-12-00781-s001.zip › genes-1206131-supplementary.pdf]

Table S1. List of gene targets in the Oncomine Comprehensive Assay v3. (Thermo Fisher Scientific, Waltham, MA, USA)–161 gene panel.

Hotspot genes		Full-length genes	Copy number genes	Gene fusions (inter- and intragenic)
AKT1	PIK3CA	ATM	AKT1	ALK
ALK	PPP2R1A	BAP1	AR	AXL
AR	PTPN11	BRCA1	CCND1	BRAF
ARAF	RAC1	BRCA2	CCNE1	EGFR
BRAF	RAF1	CDKN2A	CDK4	ERBB2
BTK	RET	FBXW7	CDK6	ERG
CBL	RHEB	MSH2	EGFR	ETV1
CDK4	RHOA	NF1	ERBB2	ETV4
CHEK2	SF3B1	NF2	FGFR1	ETV5
CSF1R	SMO	NOTCH1	FGFR2	FGFR1
CTNNB1	SPOP	PIK3R1	FGFR3	FGFR2
DDR2	SRC	PTCH1	FGFR4	FGFR3
EGFR	STAT3	PTEN	FLT3	NTRK1
ERBB2	U2AF1	RB1	IGF1R	NTRK3
ERB83	XPO1	SMARCB1	KIT	PDGFRA
ERBB4	AKT2	STK11	KRAS	PPARG
ESR1	AKT3	TP53	MDM2	RAF1
EZH2	AXL	TSC1	MDM4	RET
FGFR1	CCND1	TSC2	MET	ROS1
FGFR2	CDK6	ARID1A	MYC	AKT2
FGFR3	ERCC2	ATR	MYCL	AR
FLT3	FGFR4	ATRX	MYCN	BRCA1
FOXL2	H3F3A	CDK12	PDGFRA	BRCA2
GATA2	HIST1H3B	CDKN1B	PIK3CA	CDKN2A
GNA11	MAP2K4	CDKN2B	PPARG	ERB84
GNAQ	MDM4	CHEK1	TERT	ESR1
GNAS	MYC	CREBBP	AKT2	FGR
HNF1A	MYCN	FANCA	AKT3	FLT3
HRAS	NTRK1	FANCD2	ALK	JAK2
IDH1	NTRK2	FANCI	AXL	KRAS
IDH2	PDGFRB	MLH1	BRAF	MDM4
JAK1	PIK3CB	MRE11A	CCND2	MET
JAK2	ROS1	MSH6	CCND3	MYB
JAK3	SMAD4	NBN	CDK2	MYBL1
KDR	TERT	NOTCH2	CDKN2A	NF1
KIT	TOP1	NOTCH3	CDKN2B	NOTCH1
KNSTRN		PALB2	ESR1	NOTCH4
KRAS		PMS2	FGF19	NRG1
MAGOH		POLE	FGF3	NTRK2
MAP2K1		RAD50	NTRK1	NUTM1
MAP2K2		RAD51	NTRK2	PDGFRB
MAPK1		RAD51B	NTRK3	PIK3CA
MAX		RAD51C	PDGFRB	PRKACA
MED12		RAD51D	PIK3CB	PRKACB
MET		RNF43	RICTOR	PTEN
MTOR		SETD2	TSC1	RAD51B
MYD88		SLX4	TSC2	RB1
NFE2L2		SMARCA4		RELA
NRAS				RSPO2
PDGFRA				RSPO3
				TERT

References

https://assets.thermofisher.com/TFS-Assets/LSG/brochures/oncomine-comprehensive-assay-v3-flyer.pdf
